# Serological inference of past primary and secondary dengue infection: implications for vaccination

**DOI:** 10.1098/rsif.2019.0207

**Published:** 2019-07-31

**Authors:** Ha Minh Lam, Huynh Thi Phuong, Nguyen Ha Thao Vy, Nguyen Thi Le Thanh, Pham Ngoc Dung, Thai Thi Ngoc Muon, Nguyen Van Vinh Chau, Isabel Rodríguez-Barraquer, Derek A. T. Cummings, Bridget A. Wills, Maciej F. Boni, Maia A. Rabaa, Hannah E. Clapham

**Affiliations:** 1 Oxford University Clinical Research Unit, Wellcome Trust Major Overseas Programme, Ho Chi Minh City, Vietnam; 2 Centre for Tropical Medicine and Global Health, Nuffield Department of Medicine, University of Oxford, Oxford, UK; 3 Laboratory Department, An Giang Central General Hospital, An Giang, Vietnam; 4 Department of Biochemistry, Quang Ngai General Hospital, Quang Ngai, Vietnam; 5 Hospital for Tropical Diseases, Ho Chi Minh City, Vietnam; 6 Department of Medicine, University of California, San Francisco, CA, USA; 7 Department of Biology, University of Florida, Gainesville, FL, USA; 8 Emerging Pathogens Institute, University of Florida, Gainesville, FL, USA; 9 Department of Biology, Center for Infectious Disease Dynamics, Pennsylvania State University, University Park, PA, USA

**Keywords:** dengue, Vietnam, serostatus, vaccination, force of infection, IgG antibody

## Abstract

Owing to the finding that Dengvaxia^®^ (the only licensed dengue vaccine to date) increases the risk of severe illness among seronegative recipients, the World Health Organization has recommended screening individuals for their serostatus prior to vaccination. To decide whether and how to carry out screening, it is necessary to estimate the transmission intensity of dengue and to understand the performance of the screening method. In this study, we inferred the annual force of infection (FOI; a measurement of transmission intensity) of dengue virus in three locations in Vietnam: An Giang (FOI = 0.04 for the below 10 years age group and FOI = 0.20 for the above 10 years age group), Ho Chi Minh City (FOI = 0.12) and Quang Ngai (FOI = 0.05). In addition, we show that using a quantitative approach to immunoglobulin G (IgG) levels (measured by indirect enzyme-linked immunosorbent assays) can help to distinguish individuals with primary exposures (primary seropositive) from those with secondary exposures (secondary seropositive). We found that primary-seropositive individuals—the main targets of the vaccine—tend to have a lower IgG level, and, thus, they have a higher chance of being misclassified as seronegative than secondary-seropositive cases. However, screening performance can be improved by incorporating patient age and transmission intensity into the interpretation of IgG levels.

## 1. Introduction

Dengue is the most rapidly spreading mosquito-borne viral disease in humans [1]. Globally, this acute systemic viral infection causes around 58.4 million symptomatic cases, leading to an estimate of 10 000–20 000 deaths annually [2–4]. Although dengue virus (DENV) infections are often asymptomatic, infection can cause a wide range of clinical outcomes, from mild fever—normally followed by a full recovery—to potentially deadly dengue shock syndrome [5]. Four serotypes of dengue virus co-circulate in various parts of the world [6]. While infection with one serotype results in long-term immunity against that serotype, it is thought to induce only short-lived cross-protection against the other serotypes [5,7,8]. After this brief period of cross-protection, subneutralizing concentrations of pre-existing heterologous antibodies—generated as a result of the first exposure to dengue (primary infection)—may favour viral uptake into cells and enhance viral replication, leading to more severe symptoms during a subsequent infection with a different serotype (secondary infection) [9–11]. This infection-enhancing phenomenon is referred to as the antibody-dependent enhancement [9,12–15]. After the secondary infection, individuals are believed to be generally protected against all serotypes since post-secondary infections have rarely been observed as hospitalized cases [16–18].

To date, the only licensed dengue vaccine, Dengvaxia^®^ (CYD-TDV), is a live attenuated recombinant tetravalent vaccine that has been approved for use in individuals aged 9–45 years with previous documented exposure to dengue [19,20]. The efficacy of this vaccine varies by dengue serotype, age at vaccination and serostatus prior to vaccination [21,22]. While high efficacy (up to 93.7%) was reported in individuals who were seropositive prior to vaccination [21], a study has shown that the vaccine increases the risk of developing severe illness among seronegative recipients [22]. Therefore, the World Health Organization (WHO) has recommended a ‘pre-vaccination screening strategy’ for Dengvaxia^®^, in which only seropositive individuals are vaccinated [23,24]. However, in order to decide whether and how to carry out individual screening, it is useful to estimate the force of infection (FOI; a measurement for the transmission intensity, quantifying the hazard of infection that susceptible individuals experience) of dengue in individual populations [23,25]. In general, mass vaccination programmes are less likely to cause increased hospitalization risk in places where the transmission intensity is higher [26,27]. Yet, in hyperendemic settings, the cost-effectiveness of vaccination may decrease substantially, as many vaccine doses could be given to individuals who are already naturally protected by a prior secondary infection. Furthermore, since the FOI of dengue is not a static quantity [28], dengue seroprevalence and screening performance may change over time, requiring re-evaluation of the cost–benefit of vaccination. Therefore, understanding the dynamics of dengue FOI is vital for designing, implementing and monitoring vaccine interventions [28].

Indirect enzyme-linked immunosorbent assays (ELISAs) to detect anti-dengue immunoglobulin G (IgG) antibodies have often been employed to infer individual infection histories and to understand the population-level exposure to dengue, through which the FOI of dengue can be estimated [29–31]. Conventionally, interpretation of these assays uses a single cut-off point with which ELISA antibody levels are binarily interpreted to be either seronegative or seropositive. The sensitivity and specificity of these tests depend on the specific cut-off point used [32]. Since performance of the cut-off can be population specific, re-calibration is recommended for each new population [31]. However, calibration can be impractical in some settings as it requires sets of samples with well-characterized exposure history; therefore, the same cut-off is frequently used across different populations, potentially reducing the accuracy of interpretation. When put into context of dengue vaccination, this loss of accuracy may lead to seronegative individuals being vaccinated or seropositive cases not being vaccinated [23] and hence leading to loss in vaccination effectiveness and even increases in hospitalization rates.

Approximately 10.5 million dengue virus infections occur every year in Vietnam, a quarter of which are symptomatic cases [2]. This high endemicity makes the country an ideal site for vaccine trials [33] and a setting that could benefit from a routine use of an effective dengue vaccine. Furthermore, the fact that dengue transmission intensity varies greatly across the country—with higher incidence observed in southern provinces [34–36]—potentially enables investigation of how different transmission settings affect the implementation of vaccination programmes. As Vietnam shares common epidemiological characteristics with other countries in the region [37–39], findings from Vietnam will contribute to the global initiative for controlling dengue.

In this paper, we have two primary aims. Our first aim is to estimate the FOI of dengue and to understand how that FOI varies across different age/time periods and across different regions of Vietnam. We analysed the serological data collected from three populations of Vietnam: An Giang, Ho Chi Minh City and Quang Ngai. The inferred transmission intensities from this study will help to evaluate the potential for routine vaccination in each location [40]. The second aim of our analysis is to develop a statistical method to assess whether it is possible at a population level to use IgG levels (as measured using an indirect IgG ELISA) to differentiate past primary dengue virus infection from secondary infection [41]. This could guide the use of screening programmes to target vaccination programmes.

## 2. Material and methods

### 2.1. Serological samples

The serum samples tested in this analysis were collected as part of a serum bank established in 2009, which comprises residual serum samples from 10 hospital laboratories in 10 different provinces and cities of Vietnam. For this serum bank, 200 residual serum samples from across all age groups are randomly collected from each of the participating hospitals every 2 or 4 months. Samples were collected from both inpatients and outpatients (HIV patients are excluded) regardless of the reason for the patients' presentation at the hospitals. Samples were anonymized and delinked before being sent to the serum bank. Data on age, gender, date of sample collection and hospital ward were preserved. The serum bank was approved by the Scientific and Ethics Committee of the Hospital for Tropical Diseases in Ho Chi Minh City and the Oxford Tropical Research Ethics Committee at the University of Oxford and has been used for past sero-epidemiology studies on avian influenza [42], human influenza [43], hepatitis E virus [44] and chikungunya virus [45].

**Figure 1.**
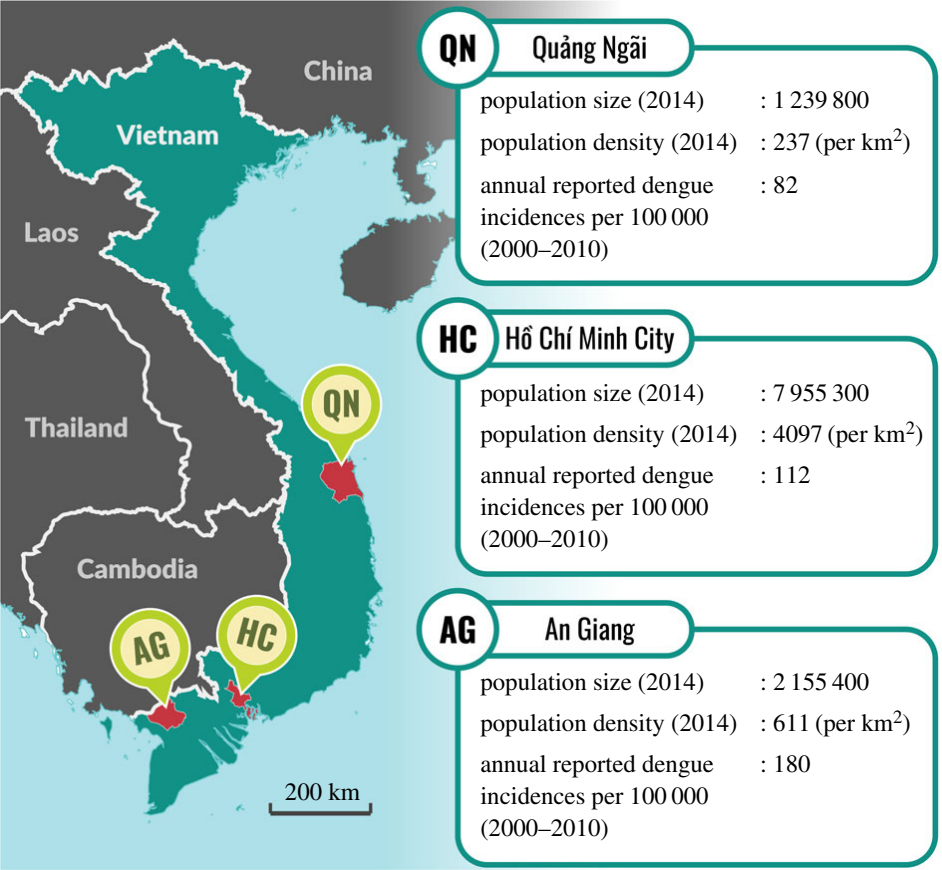
Geographical locations and population demographics of An Giang (AG), Ho Chi Minh City (HC) and Quang Ngai (QN). The demographic statistics were taken from the population census of 2014, published by the General Statistics Office (GSO) of Vietnam. The annual reported dengue incidence rates were calculated based on the number of cases reported between 2000 and 2010 [39] and the population sizes in 2009 (published by the GSO). (Online version in colour.)

For the current analysis, we randomly selected serum samples that were collected between 2014 and 2015 from the following three hospitals (figure 1): the Hospital for Tropical Diseases (Ho Chi Minh City, 356 samples), the An Giang Central General Hospital (Long Xuyen, An Giang Province, 276 samples) and the Quang Ngai General Hospital (Quang Ngai City, Quang Ngai Province, 266 samples). The serum samples were selected based on their age groups (5 year increments) with an aim of having a uniform distribution of samples across age groups (table 1). However, in Ho Chi Minh City, more samples were collected for the age groups between 10 and 25 years as they were part of a pilot analysis to assess the feasibility of using IgG levels for classifying primary and secondary exposures. Because we expected a lower dengue transmission intensity in Quang Ngai [35,36], the targeted age groups in this location were increased up to 40 (instead of 35 as in An Giang and Ho Chi Minh City) to capture more seropositive cases.

**Table 1.** The number of samples used in this analysis, categorized by age group and location. The open square brackets and the closed round brackets in the ‘age group’ column indicate that the ranges are left-inclusive and right-exclusive.

	Vietnam			
age group	An Giang	Ho Chi Minh City	Quang Ngai	Chennai, India
[00, 05)	40	43	34	0
[05, 10)	48	42	34	109
[10, 15)	47	74	30	112
[15, 20)	42	91	36	108
[20, 25)	43	52	29	99
[25, 30)	44	45	31	115
[30, 35)	12	9	38	118
[35, 40)	0	0	34	107
[40, 45)	0	0	0	31
all	276	356	266	799

### 2.2. Serological assays

Each of the selected serum samples was tested using a Panbio Dengue IgG Indirect ELISA kit (Standard Diagnostics, Inc., Gyeonggi-do, Republic of Korea) according to the manufacturer's instructions [46].

As per the manufacturer's instructions, the concentrations of IgG antibodies in each sample were quantified via measurement of optical density (OD). An *OD cut-off value* was calculated at the end of each experiment by multiplying the average OD of the triplicate of the *calibrator* (one of the control samples of the ELISA kit) with the calibration factor (specified for each kit by the manufacturer). The IgG level, *Y_i_* (measured in *Panbio units*), of each individual, *i*, was calculated as follows:

$$Y_{i}=\frac{\mathrm{OD}\;\mathrm{of}\;\mathrm{sample}\;i}{\mathrm{OD}\;\mathrm{cut}\text{-}\mathrm{off}\;\mathrm{value}}\times 10.$$

### 2.3. Statistical models

As our samples are all from one time point, it is not possible to separate the effects of age from those of time on the FOI of dengue [47]. In this paper, we chose to present heterogeneous FOIs as if they were age specific; however, it is also possible to interpret this heterogeneity as time-dependent FOIs (§4).

#### 2.3.1. Binary models

We first estimated the FOI of dengue using a series of simple catalytic models [47], which we refer to as binary models. In these models, we interpreted the IgG level of each individual as either seronegative or seropositive. Although the manufacturer of the ELISA kits suggested using a double cut-off system to interpret the antibody levels (i.e. a sample, *i*, is considered seronegative when the IgG level *Y_i_* < 9, and seropositive when *Y_i_* > 11), we simplified the interpretation by setting a single cut-off at 10 (samples are seronegative when *Y_i_* < 10 and seropositive when *Y_i_* ≥ 10). The effect of this simplification on our results should be minimal as few samples in our dataset fall into the recommended equivocal range between 9 and 11 (electronic supplementary material, figure S1).

Let *E_i_* denote the number of dengue serotypes by which individual *i* has been challenged. The probability that the individual is still naive to dengue is $P(E_{i}=0)=e^{-{\bar{E}}_{i}}$, in which ${\bar{E}}_{i}$ is calculated as follows [47]:

$$\begin{array}{rl} {\bar{E}}_{i} & =\sum_{u=1}^{p}1[\kappa_{u}<a_{i}]\times (\kappa_{u}-\kappa_{u-1})\times \lambda_{\kappa_{u-1},\kappa_{u}}\\ & \;+\left[a_{i}-\underset{\kappa_{v}}{\max}(\kappa_{v}<a_{i})\right]\times \lambda_{\kappa_{v},\kappa_{v+1}}, \end{array}$$

where *a_i_* is the age (in years) of individual *i*; $\lambda_{\kappa_{u-1},\kappa_{u}}$ is the total annual FOI of all the four serotypes of dengue that the age group between *κ_u_*_−1_ and *κ_u_* years are exposed to; *κ_u_*, *κ_u_*_−1_ are age cut-offs (*κ*_0_ = 0 and *κ_p_* = ∞); *p* ∈ {1, 2, 3} is the number of age-specific FOIs in the considered model; and **1**[*κ_u_* < *a_i_*] is an indicator function, i.e.

$$1[\kappa_{u}<a_{i}]=\{\begin{array}{cc} 1 & \mathrm{if}\;\kappa_{u}<a_{i}\\ 0 & \mathrm{otherwise}. \end{array}$$

The binomial likelihoods of the binary models were computed as follows:

$$L(\theta |Y)\propto \overset{n}{\underset{i=1}{\prod }}[1[Y_{i}<10]\times e^{-{\bar{E}}_{i}}+1[Y_{i}\geq 10]\times (1-e^{-{\bar{E}}_{i}})],$$

where ***θ*** denotes all the parameters in the models, including *κ*_0_,…, *κ_p_* and $\lambda_{\kappa_{0},\kappa_{1}},\ldots ,\lambda_{\kappa_{\;p-1},\kappa_{p}}$; *n* is the number of samples in our dataset; and

$$1[x]=\{\begin{array}{cc} 1 & \mathrm{if}\;x\;\mathrm{is}\;\mathrm{true}\\ 0 & \mathrm{otherwise}. \end{array}$$

#### 2.3.2. Continuous models

In continuous models, we assumed that the observed ELISA antibody levels of individuals depend on the number of exposures to dengue (*E_i_*). The antibody levels of seropositive individuals were modelled by *γ* distributions. When individuals are seronegative, their IgG levels are expected to be close to zero; therefore, the IgG levels of this group were assumed to be exponentially distributed.

In models with two distributions of IgG levels (i.e. *Y_i_*|*E_i_* = 0 and *Y_i_*|*E_i_* ≥ 1), the binomial likelihoods of the continuous models were calculated as follows:

$$L(\theta |Y)\propto \overset{n}{\underset{i=1}{\prod }}[\;f(Y_{i}|E_{i}=0)\times e^{-{\bar{E}}_{i}}+f(Y_{i}|E_{i}\geq 1)\times (1-e^{-{\bar{E}}_{i}})],$$

where *f*(*Y_i_*|*E_i_* = 0) and *f*(*Y_i_*|*E_i_* ≥ 1) are the probability density functions of IgG levels of seronegative and seropositive individuals, respectively.

Similarly, in models with three IgG-level distributions (i.e. *Y_i_*|*E_i_* = 0, *Y_i_*|*E_i_* = 1 and *Y_i_*|*E_i_* ≥ 2), the multinomial likelihoods were calculated as follows:

$$\begin{array}{rl} L(\theta |Y)\propto \overset{n}{\underset{i=1}{\prod }} & [\;f(Y_{i}|E_{i}=0)\times e^{-{\bar{E}}_{i}}+f(Y_{i}|E_{i}=1)\\ & \;\times 4e^{-\frac{3}{4}{\bar{E}}_{i}}\left(1-e^{-\frac{1}{4}{\bar{E}}_{i}}\right)+f(Y_{i}|E_{i}\geq 2)\\ & \;\times \left(1-e^{-{\bar{E}}_{i}}-4e^{-\frac{3}{4}{\bar{E}}_{i}}\left(1-e^{-\frac{1}{4}{\bar{E}}_{i}}\right)\right)], \end{array}$$

where *f*(*Y_i_*|*E_i_* = 0), *f*(*Y_i_*|*E_i_* = 1) and *f*(*Y_i_*|*E_i_* ≥ 2) are the probability density functions of IgG levels of individuals given that they were estimated to be seronegative (naive to dengue), primary seropositive (having experienced only primary infections) or secondary seropositive (having experienced secondary infections), respectively.

In this analysis, we assumed that all dengue serotypes had equal FOIs (i.e. *λ*/4) and that monotypic re-exposure did not alter IgG antibody levels.

Although it is expected that IgG levels of secondary-seropositive individuals are higher than those of primary-seropositive cases [41], we did not impose this restriction on our models.

#### 2.3.3. Model fitting and model selection

This modelling analysis was divided into two major steps. In the first step, the data from An Giang, Ho Chi Minh City and Quang Ngai were analysed separately to estimate the FOIs of dengue and to assess the likelihood of age-varying FOIs in each of the locations. Five binary models and 10 continuous models, with either constant or age-varying FOI, were tested for each population. In age-varying FOI models, the age cut-offs were either estimated or fixed at 6 years (for models with two or three age groups) and 18 years (for models with three age groups). In the continuous models, the number of distributions of IgG levels was either two or three.

In the second step, we assessed whether the distributions of IgG levels were shared across populations by fitting four additional continuous models to the whole Vietnamese dataset (which included all samples from An Giang, Ho Chi Minh City and Quang Ngai). In these models, the configuration of the FOI in each population and the number of IgG-level distributions were based on the best-fitting continuous models acquired from the first step of this analysis. While the baseline model of this group allowed all three IgG-level distributions to be population specific, the other models tested one of the following three assumptions: (i) all the IgG-level distributions were shared across the populations, (ii) only the IgG-level distribution of seronegative cases was population specific, or (iii) only those of seropositive cases were population specific.

The models were fitted to the data using the Metropolis–Hastings algorithm. The parameters of each model were estimated through a Markov chain Monte Carlo (MCMC) of 800 000 iterations (with the first 300 000 iterations removed as burn-in). Model convergence was visually assessed. Models were compared using the deviance information criterion (DIC). Models were classified as ‘best fitting’ if their DICs were within a 10-unit difference compared with the lowest DIC of the corresponding model group. The likelihood function and chain iteration were implemented in a C++ application, whose source code is accessible at https://gitlab.com/lamhm/igg-to-foi. Post-MCMC analyses were carried out in R [48].

#### 2.3.4. Estimating seroprevalence

The dengue seroprevalence of a group of individuals of a given age, *a*, was calculated using the relationship between age and probability of exposure, established in the catalytic model [47],

$$\begin{array}{r} \mathrm{Prev}(a)=1-\exp\;(-\sum_{u=1}^{p}1[\kappa_{u}<a]\times (\kappa_{u}-\kappa_{u-1})\times \lambda_{\kappa_{u-1},\kappa_{u}}\\ +\left[a-\underset{\kappa_{v}}{\max}(\kappa_{v}<a)\right]\times \lambda_{\kappa_{v},\kappa_{v+1}}), \end{array}$$

where the values of the FOIs (*λ*'s) were drawn from the posteriors of the tested models.

#### 2.3.5. Estimating primary-seropositive probability based on IgG-level distributions

To demonstrate the potential of using the IgG-level distributions (inferred from the continuous models) in guiding who should be vaccinated, we built a table for each of the study populations to show the probability that individuals of particular age groups with certain IgG levels were primary seropositive. Each probability was calculated as follows:

$$\begin{array}{rl} P(E_{i}=1|Y_{i}) & =\left[\;f(Y_{i}|E_{i}=1)\times 4e^{-\frac{3}{4}{\bar{E}}_{i}}\left(1-e^{-\frac{1}{4}{\bar{E}}_{i}}\right)\right]\\ & \;÷\left[\;f(Y_{i}|E_{i}=0)\times e^{-{\bar{E}}_{i}}+f(Y_{i}|E_{i}=1\right)\\ & \;\times 4e^{-\frac{3}{4}{\bar{E}}_{i}}(1-e^{-\frac{1}{4}{\bar{E}}_{i}})+f(Y_{i}|E_{i}\geq 2)\\ & \;\times (1-e^{-{\bar{E}}_{i}}-4e^{-\frac{3}{4}{\bar{E}}_{i}}(1-e^{-\frac{1}{4}{\bar{E}}_{i}}))], \end{array}$$

where *f*(*Y_i_*|*E_i_* = 0), *f*(*Y_i_*|*E_i_* = 1) and *f*(*Y_i_*|*E_i_* = 2) are, respectively, the probability density functions of the seronegative, the primary-seropositive and the secondary-seropositive IgG-level distributions; and the variable ${\bar{E}}_{i}$ can be calculated when the age of the sample and the FOI are given.

The FOIs and the IgG-level distributions used for this demonstration were based on the median estimates from the best-fitting continuous models.

### 2.4. Testing continuous models on a non-Vietnamese population

To assess the performance of our method on a different population, we tested our continuous models on a serological dataset of 799 samples collected from Chennai, India, in 2011 (table 1). The details of these samples have been presented elsewhere [29].

It is noteworthy that this dataset contains only samples from individuals older than 5 years, making it impossible to infer age-specific FOIs for young children. Furthermore, since the population of Chennai was exposed to a very high annual FOI of dengue (0.23 between 2004 and 2011 and 0.10 before 2004) [29], it is likely that the majority of the cases in this dataset had experienced a secondary exposure. This was presumably the main cause of the lack of samples with low or moderate IgG levels in the dataset, thus rendering it difficult to infer the IgG-level distributions for seronegative and primary cases. To overcome this issue, we used the posteriors of the IgG-level distributions of seronegative and primary-seropositive cases from a Vietnamese population (inferred from the previous analysis) as strong priors for the corresponding IgG-level distributions of Chennai.

Six continuous models with three IgG-level distributions (as the analysis on the Vietnamese populations strongly supported three IgG-distribution models; see §3) were fitted to the data from Chennai. The FOI in Chennai was considered as being either constant or time varying (note that we interpret the heterogeneity of the FOI in Chennai as being time dependent as suggested in the original paper). The time cut-offs were either estimated or set at 6, 7 and 18 years (before 2011). The 7 year cut-off was added into this analysis based on the findings of the original paper [29]. Each of the models was fitted through an removed as burn-in.

## 3. Results

Eight of 15 binary models that were tested on the data obtained from the Vietnamese populations resulted in converged chains (electronic supplementary material, table S1). Six of the seven unconverged models had age-varying FOI with estimated age cut-offs. The other unconverged model (*AgiBin04*), although having fixed age cut-offs, could not estimate the FOI for the age group of above 18 years in An Giang since the seroprevalence of this age group may have been saturated at nearly 100%.

Fitting the continuous models to the data from An Giang, Ho Chi Minh City and Quang Ngai separately led to 19 converged chains (electronic supplementary material, table S2). All of the 11 models that did not converge had issue estimating the age cut-offs for age-varying FOIs. For all three populations, models with three distributions for IgG levels significantly improved the goodness of fit compared with models with only two IgG-level distributions. All of the four continuous models that were fitted to the whole Vietnamese dataset converged.

### 3.1. Force of infection of dengue in the Vietnamese population

All the best-fitting models suggest that, in the long term, individuals in An Giang and Ho Chi Minh City experienced a significantly higher risk of infection than those in Quang Ngai (table 2 and electronic supplementary material, table S1). In comparison with the binary models, the continuous models tended to result in slightly lower FOI estimates for An Giang and higher approximations for Ho Chi Minh City and Quang Ngai.

**Table 2.** Median estimates of the annual forces of infection of dengue from the best-fitting continuous models that were fitted on the data of An Giang, Ho Chi Minh City or Quang Ngai separately. Numbers in brackets represent the 95% credible intervals of the estimates. All of the listed models support three IgG-level distributions.

		age groups		force of infection^b^		
	model (DIC)	count	cut-offs^a^	first age group	second age group	third age group
An Giang	AgiCon33 (1936)	2	9.60 (7.12; 14.08)	0.04 (0.02; 0.07)	0.20 (0.15; 0.29)	—
	AgiCon32 (1941)	2	6	0.04 (0.02; 0.06)	0.15 (0.12; 0.18)	—
	AgiCon34 (1943)	3	6 and 18	0.04 (0.02; 0.07)	0.14 (0.10; 0.18)	0.20 (0.09; 0.37)
Ho Chi Minh City	HcmCon31 (2431)	1	—	0.12 (0.11; 0.14)	—	—
	HcmCon32 (2431)	2	6	0.10 (0.06; 0.15)	0.14 (0.11; 0.17)	—
	HcmCon34 (2432)	3	6 and 18	0.09 (0.05; 0.14)	0.15 (0.11; 0.20)	0.10 (0.02; 0.19)
Quang Ngai	QngCon31 (1727)	1	—	0.05 (0.04; 0.06)	—	—
	QngCon32 (1727)	2	6	0.08 (0.04; 0.12)	0.04 (0.03; 0.06)	—
	QngCon34 (1727)	3	6 and 18	0.09 (0.05; 0.14)	0.03 (0.00; 0.06)	0.06 (0.03; 0.09)

^a^The age cut-off in the model AgiCon33 was estimated by the MCMC run. In the other models, the age cut-offs (if applicable) were fixed*.*

^b^In models with age-varying force of infection, the first age group corresponds to the youngest age group.

The best-fitting models for An Giang unequivocally suggest an age-varying FOI. In other words, individuals in An Giang showed a low FOI of 0.06 (95% credible interval (CI): 0.04–0.09, in *AgiBin02*) or 0.04 (95% CI: 0.02–0.07, in *AgiCon33*) prior to the age of 6 years (*AgiBin02*) or 9.6 years (*AgiCon33*) and experienced a much higher FOI of 0.17 (95% CI: 0.13–0.21, in *AgiBin02*) or 0.20 (95% CI: 0.15–0.29, in *AgiCon33*) after that. One of the continuous models (*AgiCon34*) indicates that individuals in An Giang may have been exposed to the highest FOI after they reached 18 years; however, the difference between the FOI estimates of the above 18 years age group and that of the 6–18 years age group is not statistically significant.

Meanwhile, the results for Ho Chi Minh City and Quang Ngai suggest that the FOIs of dengue in these locations were likely to be constant. Although some of the best-fitting models for Ho Chi Minh City and Quang Ngai also suggest age-varying FOIs, the FOI estimates in these models varied little (not statistically significant) across age groups. In Ho Chi Minh City, the FOI was around 0.07 (95% CI: 0.06–0.09, in *HcmBin01*) or 0.12 (95% CI: 0.11–0.14, in *HcmCon31*). In Quang Ngai, the estimate was 0.03 (95% CI: 0.02–0.04, in *QngBin01*) or 0.05 (95% CI: 0.11–0.14, in *QngCon31*).

### 3.2. Seroprevalence of dengue in the Vietnamese population

**Figure 2.**
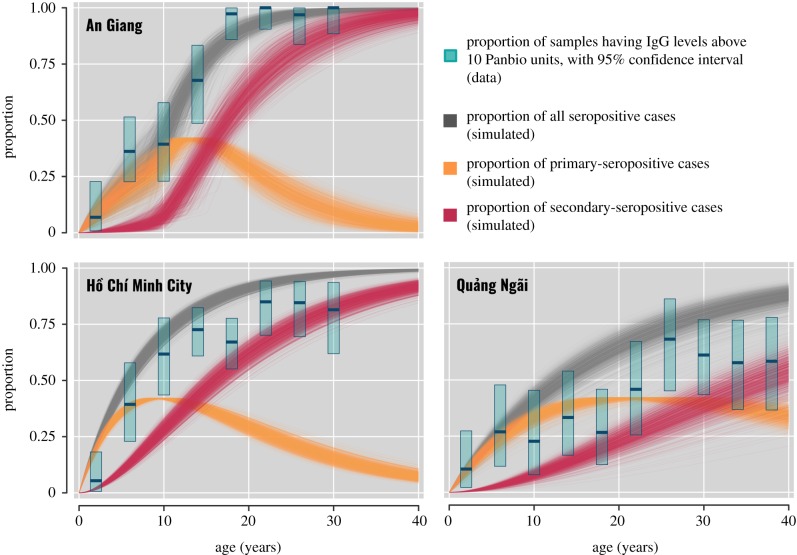
The seroprevalence of dengue in the study populations. The bars represent the proportion (with 95% confidence intervals) of samples in each age group of 4 years that have IgG levels above 10 Panbio units. The lines show the estimates of seroprevalence from 1000 simulations, of which the parameters were drawn from the posteriors of the following models: AgiCon33 (for An Giang), HcmCon31 (for Ho Chi Minh City) and QngCon31 (for Quang Ngai). (Online version in colour.)

The seroprevalences of dengue in An Giang, Ho Chi Minh City and Quang Ngai were inferred based on the FOI estimates from the models with the lowest DIC (table 3 and figure 2). Compared with binary models, continuous models gave lower seroprevalence estimates for An Giang, but resulted in higher estimates for Ho Chi Minh City and Quang Ngai. The binary models estimated that 57.2% (95% CI: 50.6%–64.2%, in *AgiBin02*) of 9 year olds (the recommended age group for Dengvaxia) in An Giang were dengue seropositive, while only 49.0% (95% CI: 44.5%–53.6%, in *HcmBin01*) in Ho Chi Minh City and 23.4% (95% CI: 19.6%–27.2%, in *QngBin01*) in Quang Ngai were seropositive. Nevertheless, results from the continuous models suggest that Ho Chi Minh City had the highest seroprevalence among populations with 66.8% (95% CI: 62.2%–70.9%, in *HcmCon31*) of 9-year-olds being seropositive; meanwhile, the number was 34.7% (95% CI: 21.4%–49.5%, in *AgiCon33*) for An Giang and 37.2% (95% CI: 30.8%–43.5%, in *QngCon31*) for Quang Ngai. In general, the median ages of infection in An Giang (*AgiBin02*: 8.1 years; *AgiCon33*: 11.0 years) and Ho Chi Minh City (*HcmBin01*: 9.3 years; *HcmCon31*: 5.7 years) are substantially lower than the estimate for Quang Ngai (*QngBin01*: 23.4 years; *QngCon31*: 13.4 years).

**Table 3.** Estimates of the median age of infection and the proportion of 9-year-olds being seropositive. The numbers were approximated by the following models: AgiBin02 and AgiCon33 (for An Giang), HcmBin01 and HcmCon31 (for Ho Chi Minh City) and QngBin01 and QngCon31 (for Quang Ngai). Numbers in brackets represent the 95% credible intervals of the estimates. In these estimates, the FOIs of Ho Chi Minh City and Quang Ngai were assumed to be constant over age and time. Meanwhile, the FOI of An Giang was assumed to be age dependent (rather than time dependent) with an age cut-off fixed at 6 years (in the binary model, AgiBin02) or estimated at 9.6 years (in the continuous model, AgiCon33).

	binary model		continuous model	
population	median age of infection	seroprevalence at 9 years old (%)	median age of infection	seroprevalence at 9 years old (%)
An Giang	8.1 (7.0; 8.9)	57.2 (50.6; 64.2)	11.0 (9.1; 12.9)	34.7 (21.4; 49.5)
Ho Chi Minh City	9.3 (8.1; 10.6)	49.0 (44.5; 53.6)	5.7 (5.1; 6.4)	66.8 (62.2; 70.9)
Quang Ngai	23.4 (19.7; 28.5)	23.4 (19.6; 27.2)	13.4 (10.9; 16.9)	37.2 (30.8; 43.5)

### 3.3. IgG-level distributions from continuous models

**Figure 3.**
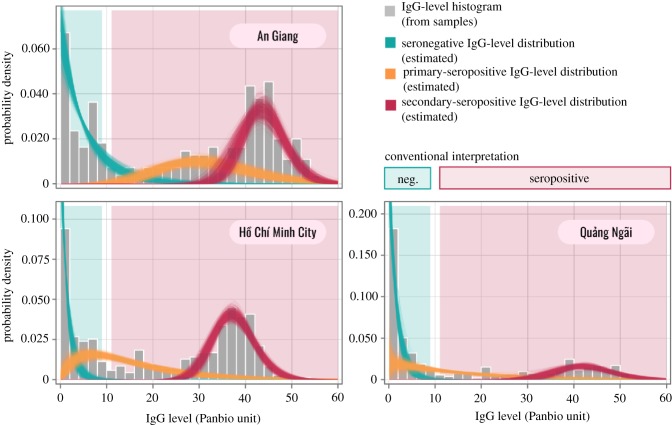
The histogram of the measured IgG levels (bars) and the inferred IgG-level distributions (lines) of the three exposure classes (seronegative, primary seropositive and secondary seropositive) across the study populations. The IgG-level distributions were simulated 1000 times based on the posteriors of the following models: AgiCon33 (for An Giang), HcmCon31 (for Ho Chi Minh City) and QngCon31 (for Quang Ngai). (Online version in colour.)

All the best-fitting continuous models acquired from analysing the data of each population separately suggest three distributions for IgG levels (table 4 and figure 3). These distributions, in order of increasing mean values, correspond to three exposure classes: dengue-naive individuals (seronegative), individuals with primary exposures (primary seropositive) and individuals with secondary exposures (secondary seropositive). In all three populations, the IgG levels of secondary-seropositive cases were well separated from those of seronegative cases. However, primary IgG levels and seronegative IgG levels are less distinguishable—especially in Ho Chi Minh City and Quang Ngai—as the two distributions overlap. An Giang stands out with the mean IgG levels of seronegative cases (*AgiCon33*: 5.3 (95% CI: 4.1–7.1)) and primary cases (32.4 (95% CI: 28.4–36.2)) notably higher than those in Ho Chi Minh City (*HcmCon31*: 1.3 for seronegative individuals, 14.9 for primary individuals) and Quang Ngai (*QngCon31*: 1.6 for seronegative individuals and 15.1 for primary individuals). The secondary IgG distribution for An Giang (mean: 43.8; standard deviation (s.d.): 4.7), however, closely resembles that of Quang Ngai (mean: 42.4; s.d.: 5.9) and has a slightly higher mean than that of Ho Chi Minh City (mean: 37.5; s.d.: 4.5).

**Table 4.** Median estimates of the mean and the standard deviation (s.d.) of the IgG-level distributions inferred from the best-fitting continuous models that were fitted on the data of An Giang, Ho Chi Minh City or Quang Ngai separately. Numbers in brackets represent the 95% highest probability density range of the estimates. In the models below, all IgG-level distributions were estimated for each population separately.

			primary seropositive		secondary seropositive	
	model (DIC)	mean and s.d. of seronegative	mean	s.d.	mean	s.d.
An Giang	AgiCon33 (1936)	5.3 (4.1; 7.1)	32.4 (28.4; 36.2)	10.1 (7.9; 13.1)	43.8 (42.5; 45.0)	4.7 (3.7; 6.0)
	AgiCon32 (1941)	5.3 (4.1; 7.1)	32.4 (28.5; 36.1)	10.0 (7.8; 12.8)	44.1 (42.8; 45.2)	4.5 (3.7; 5.7)
	AgiCon34 (1943)	5.3 (4.0; 7.0)	32.3 (28.3; 36.1)	10.0 (7.8; 13.1)	43.9 (42.6; 45.1)	4.7 (3.7; 5.9)
Ho Chi Minh City	HcmCon31 (2431)	1.3 (1.0; 1.8)	14.9 (11.9; 18.5)	10.8 (8.1; 14.0)	37.5 (36.7; 38.3)	4.5 (3.9; 5.3)
	HcmCon32 (2431)	1.3 (1.0; 1.8)	15.0 (12.0; 18.6)	10.8 (8.1; 14.0)	37.5 (36.7; 38.3)	4.5 (3.9; 5.3)
	HcmCon34 (2432)	1.3 (1.0; 1.8)	14.9 (11.9; 18.5)	10.7 (8.1; 13.9)	37.5 (36.7; 38.3)	4.6 (3.9; 5.3)
Quang Ngai	QngCon31 (1727)	1.6 (1.2; 2.1)	15.1 (10.3; 21.2)	15.8 (10.6; 23.9)	42.4 (40.4; 44.3)	5.9 (4.4; 7.9)
	QngCon32 (1727)	1.5 (1.1; 2.0)	14.5 (9.3; 22.0)	14.3 (9.2; 20.4)	42.4 (40.4; 44.3)	6.0 (4.5; 8.1)
	QngCon34 (1727)	1.5 (1.1; 2.0)	14.3 (9.3; 21.4)	14.1 (9.1; 20.3)	42.3 (40.3; 44.2)	6.1 (4.6; 8.2)

The four additional continuous models designed to test the population specificity of the IgG-level distributions were configured to have (i) constant FOIs for Ho Chi Minh City and Quang Ngai, (ii) an age-varying FOI for An Giang (with two age groups), and (iii) three distributions for IgG levels (this configuration was based on *AgiCon33*, *HcmCon31* and *QngCon31*). The results from this group of models show that allowing the IgG-level distribution to be population specific substantially improved the goodness of fit (electronic supplementary material, table S2).

### 3.4. Estimating primary-seropositive probability based on IgG level and patient age

**Figure 4.**
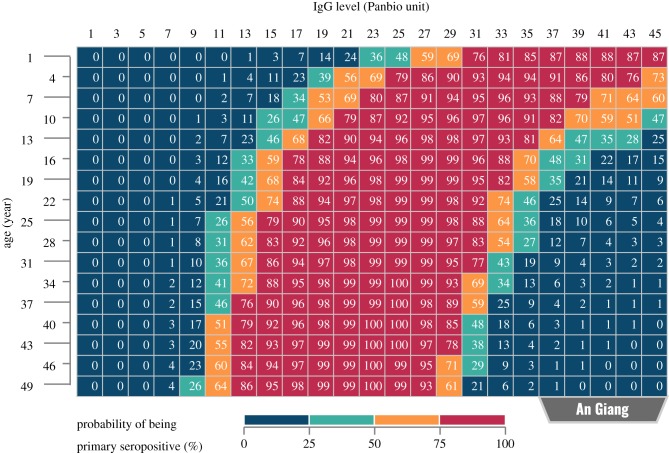
Demonstration of using a continuous model to estimate the probability of a given sample being primary seropositive. This estimation method makes use of not only the IgG levels (measured by ELISAs) but also the ages of patients and the force of infection of dengue in the population. This probability table was built based on the IgG-level distributions and the median FOI estimate of An Giang, inferred from the AgiCon33 model. The left-most area of the table (in blue) represents individuals who are likely to be seronegative. Meanwhile, individuals falling into the rightmost area (also in blue) are likely to be secondary seropositive. (Online version in colour.)

To demonstrate the potential of incorporating sample age and dengue FOI into the interpretation of IgG levels, a probability table showing the likelihood of individuals being primary seropositive was generated for each of the study populations (figure 4; electronic supplementary material, figures S2 and S3). The FOI estimates and the IgG-level distributions used for the calculation were taken from the lowest DIC continuous models: *AgiCon33* (for An Giang), *HcmCon31* (for Ho Chi Minh City) and *QngCon31* (for Quang Ngai). In general, the tables suggest that using an ELISA cut-off of 11 Panbio units (as suggested by the manufacturer of the kits) would lead to many seronegative cases in An Giang being misclassified as seropositive and many primary-seropositive cases in Ho Chi Minh City and Quang Ngai being misclassified as seronegative. Furthermore, within a population, children are more likely to be seronegative than adults if they have the same low or moderate IgG level.

### 3.5. Testing continuous models on Chennai data

Using the posteriors of Ho Chi Minh City or Quang Ngai as the priors for the seronegative and the primary-seropositive IgG-level distributions of Chennai always led to either non-convergence of the models or implausible results (e.g. the distribution mean of primary-IgG levels falling below 10 or unrealistically high FOI estimates). Therefore, in this section, we choose to present only the results from the models that used the posteriors of An Giang as the priors.

Five of the six tested continuous models for Chennai—with the posteriors of the *AgiCon33* model (the lowest DIC model for An Giang) used as the priors—resulted in converged chains (electronic supplementary material, table S3). All of the best-fitting models support a time-varying FOI in Chennai with a time cut-off of 6.4–13.3 years. According to the parameters estimated with these models, a recent period during which the FOI in Chennai was very high (*CheCon32e*: 0.28; 95% CI: 0.23–0.33) was preceded by a period with a much lower FOI (*CheCon32e:* 0.06; 95% CI: 0.04–0.08). The estimated seropositive-IgG and primary-IgG distributions of Chennai (electronic supplementary material, figure S5) closely resemble the corresponding distributions for An Giang, with the distribution means, respectively, being 4.8 (95% CI: 3.7–6.1, in *CheCon32e*) and 32.3 (95% CI: 30.3–34.4, in *CheCon32e*). Meanwhile, the distribution of secondary-IgG levels of Chennai has a slightly lower mean (*CheCon32e*: 41.1; 95% CI: 40.9–41.9) than that of An Giang (*AgiCon33*: 43.8; 95% CI: 42.5–45.0).

## 4. Discussion

In this analysis, we used a basic age seroprevalence approach to infer the FOI of dengue virus at three sites in Vietnam. Multiple models of FOI and antibody acquisition were tested, with the majority of best-fitting models agreeing that Quang Ngai had a lower overall FOI than Ho Chi Minh City or An Giang and that the FOI in An Giang was likely to be age varying, while the FOIs in Ho Chi Minh City and Quang Ngai were generally constant. In addition, we show that using a quantitative approach to IgG levels (measured by indirect ELISAs) can help to distinguish primary-seropositive samples from secondary-seropositive samples by using the relationship between age and serostatus (i.e. older individuals are more likely to be secondary seropositive in long-term endemic settings) to calibrate the relationship between IgG level and serostatus.

Although we cannot perform a direct statistical comparison between the continuous models and the binary models, it is likely that the continuous models would generally lead to more robust results. First, the continuous models do not suffer from the loss of information due to the categorization of IgG levels and, hence, may yield more precise information (e.g. the age cut-offs of An Giang could only be estimated in the continuous models). Second, the histograms of the IgG-level data from each of the populations show better fit to a mixture of three γ distributions (as in the continuous models) rather than to a categorical distribution. Finally, while the sensitivity and the specificity of the ELISA are mathematically incorporated into the continuous models (via the IgG-level distributions), these factors are not considered in the binary models; this seems to be the major reason for the discrepancy in the FOI and the seroprevalence estimates between the two categories of models.

The non-constant FOI of An Giang can be interpreted in two ways. When interpreted as time dependent, this heterogeneity would mean that the FOI of dengue in An Giang quickly decreased from 0.17–0.20 before 2005 to 0.04–0.06 after 2005 (the time cut-off is around 10 years prior to the sample collection). On the other hand, interpreting the heterogeneous FOI of An Giang as age specific would mean that individuals older than 10 years in this population were constantly exposed to very high transmission while the below 10 years age group experienced a low risk of infection, thus substantially delaying the median age of infection. Although a time-varying FOI could be caused by demographic changes or control efforts over time [28,49], it is more likely that the FOI of An Giang was age dependent for two reasons. First, according to national surveillance, the annual number of reported dengue cases in An Giang was generally stable across the years between 1994 and 2010 [39,50]. Second, interpreting the FOI as age varying in our continuous models led to seroprevalence estimates (table 3) that are consistent with the seroprevalence of 22–30% among 9-year-olds, approximated by a cohort study in Long Xuyen (the capital city of An Giang) between 2003 and 2007 [51]. However, further study is needed to confirm whether the FOI of An Giang is age or time specific as this will affect vaccination strategies. An age-varying FOI (inferred from the past data) could be used directly for vaccine recommendations in the near future. Meanwhile, a time-varying FOI would be useful only if we have adequate understanding of why and how transmission intensity changes over time.

While the binary models led to lower FOI estimates for Ho Chi Minh City and Quang Ngai, compared with the continuous models, this may be an artefact of many primary-seropositive individuals being misclassified as seronegative in the binary models due to the overlapping IgG-level distributions of primary-seropositive and seronegative cases (shown by the continuous models). Despite the difficulty of reliably comparing FOI estimates inferred from serological and case data [52], the constant FOI of Ho Chi Minh City (around 0.12) estimated from our best-fitting continuous models generally agrees with the previous findings, which used case data to infer the city's annual attack rate of dengue to be 11.6% [38]. Since this is the first study to measure the FOI of dengue in Quang Ngai, we cannot validate our estimates in this population. However, the low transmission intensity inferred by our models fits well with the low incidence rate in Quang Ngai [39] (figure 1).

The fact that our continuous models led to estimates that are consistent with the findings of other studies (which used different types of data or different methods of sample collection) indicates that sampling bias is unlikely to be a major issue in our analysis despite the sera used in this study being residual samples collected from hospitals. Furthermore, testing the continuous models on the population of Chennai resulted in mostly similar outcomes to the study from which the data were collected [29], indicating the robustness of our method. Additionally, in a preliminary analysis (data not presented), we also found that the results of our continuous models were not sensitive to the assumption of whether monotypic re-exposure did alter IgG antibody levels.

The high distribution mean of primary-IgG levels in An Giang and probably also in Chennai (although the analysis of Chennai was based on strong priors) may result from the very high transmission rates in the two locations. A possible explanation for this finding is that a primary infection would increase IgG to a moderately high level, which eventually wanes if infection does not recur [41]. In other words, high FOIs in An Giang and Chennai could reflect high proportions of individuals with homotypic boosting or recent primary infections (so that their IgG levels had not yet waned). An alternative hypothesis that could explain the differences—not only in the primary-IgG levels but also in the seronegative-IgG levels—between An Giang and the other Vietnamese populations is the possibility that the distributions of IgG levels may be age dependent (even for individuals in the same exposure class). As the age structures of seronegative and primary-seropositive cases were very different across the populations, this would lead to differences in the IgG-level distributions.

The results from our models indicate that, compared with secondary-seropositive cases, primary-seropositive individuals have a higher chance of being misclassified as seronegative. The inferred IgG-level distributions from the continuous models suggest that a secondary infection generally leads to a higher IgG level than a primary infection. In fact, this IgG-boosting phenomenon has been observed in dengue patients for many years [41]; however, only one study has quantified this at a population level [53]. Additionally, our findings are consistent with the reported sensitivities in the manual of the ELISA kits, with the sensitivities of primary and secondary cases, respectively, being 33.3% (95% CI: 23.4–44.5) and 97.9% (95% CI: 92.5–99.7) in one test and 87.0% (95% CI: 66.4–97.2) and 100% (95% CI: 96.1–100) in another test [46] (we cannot specify why the sensitivities varied greatly between these tests as the manual descriptions are vague).

The low sensitivity of the ELISA on samples from primary-seropositive individuals (as observed in practice and confirmed in our continuous models) is particularly problematic when put into context of the recommended dengue vaccination strategy. On the one hand, we could take a conservative approach to avoid vaccinating seronegative individuals, probably by increasing the cut-off for the assay. In this case, most people with primary exposures—the main targets of the vaccine—would be misclassified as being seronegative and, hence, would not receive the vaccine. Conversely, if the ELISA cut-off was reduced to increase the number of primary-seropositive recipients, the risk of vaccinating a large number of seronegative individuals would also increase. Although using double cut-offs (with an equivocal buffer between the seronegative and the seropositive ranges, as suggested by the manufacturer of our ELISA kits) may reduce the misclassification rate, it is not helpful in the context of dengue vaccination since we still have to decide whether individuals with equivocal results should receive the vaccine. It has been shown that the severity of this misclassification problem strongly depends on dengue transmission intensity, hence the need to take local FOI into account when carrying out pre-vaccination screening [23,54]. In this work, we push this idea a step further by showing that patient age is another factor that should be incorporated into the interpretation of ELISA results. In other words, by combining age, IgG level and dengue FOI, one can calculate a probability table (figure 4) to assess the likelihood of each sample being primary seropositive. Although more studies are needed to apply this method at individual levels, there is potential to use this probability table at population scales. For instance, one should consider having different ELISA cut-offs for different age groups in population serosurveillance. More specifically, higher ELISA cut-offs should be set for lower age groups to compensate for the lower likelihood of younger age groups being seropositive. Additionally, in a routine vaccination campaign, our method can be employed to estimate the number of vaccine doses not given to primary-seropositive individuals and, thus, to calculate the effectiveness of the campaign.

This work shows the potential of using IgG titre data quantitatively for refining estimates of FOIs and for enhancing the accuracy of ELISA result interpretation. Our models show robust performance across different transmission settings. These results suggest that Ho Chi Minh City may be a suitable target for routine testing and vaccination campaigns against dengue, but that Quang Ngai and An Giang may not be or may require testing and vaccination at much higher ages.

## Supplementary Material

Supplementary Information for Lam et al. (2019)

## Data accessibility

The ELISA results—measured from the Vietnamese samples in this study—are publicly accessible via: https://gitlab.com/lamhm/igg-to-foi/blob/master/data/VNM_samples.csv.

## References

[1] World Health Organization. 2019 Dengue and severe dengue. https://www.who.int/en/news-room/fact-sheets/detail/dengue-and-severe-dengue (accessed on 13 May 2019).

[2] Bhatt S et al. 2013 The global distribution and burden of dengue. Nature 496, 504–507. (10.1038/nature12060) 23563266 PMC3651993

[3] Stanaway JD et al. 2016 The global burden of dengue: an analysis from the Global Burden of Disease Study 2013. Lancet Infect. Dis. 16, 712–723. (10.1016/S1473-3099(16)00026-8) 26874619 PMC5012511

[4] World Health Organization. 2019 Dengue. http://www.searo.who.int/entity/vector_borne_tropical_diseases/data/data_factsheet/en/ (accessed 13 May 2019).

[5] Simmons CP, Farrar JJ, Van Vinh Chau N, Wills B 2012 Dengue. N. Engl. J. Med. 366, 1423–1432. (10.1056/NEJMra1110265) 22494122

[6] Messina JP et al. 2014 Global spread of dengue virus types: mapping the 70 year history. Trends Microbiol.. 22, 138–146. (10.1016/j.tim.2013.12.011) 24468533 PMC3946041

[7] Sabin AB 1952 Research on dengue during World War II 1. Am. J. Trop. Med. Hyg. 1, 30–50. (10.4269/ajtmh.1952.1.30) 14903434

[8] Anderson KB et al. 2014 A shorter time interval between first and second dengue infections is associated with protection from clinical illness in a school-based cohort in Thailand. J. Infect. Dis. 209, 360–368. (10.1093/infdis/jit436) 23964110 PMC3883164

[9] Rodenhuis-Zybert IA, Moesker B, da Silva Voorham JM, van der Ende-Metselaar H, Diamond MS, Wilschut J, Smit JM 2011 A fusion-loop antibody enhances the infectious properties of immature flavivirus particles. J. Virol. 85, 11 800–11 808. (10.1128/JVI.05237-11) PMC3209313 21880758

[10] Midgley CM et al. 2011 An in-depth analysis of original antigenic sin in dengue virus infection. J. Virol. 85, 410–421. (10.1128/JVI.01826-10) 20980526 PMC3014204

[11] Halstead SB, O'Rourke EJ 1977 Dengue viruses and mononuclear phagocytes. I. Infection enhancement by non-neutralizing antibody. J. Exp. Med. 146, 201–217. (10.1084/jem.146.1.201) 406347 PMC2180729

[12] Chan KR et al. 2014 Leukocyte immunoglobulin-like receptor B1 is critical for antibody-dependent dengue. Proc. Natl Acad. Sci. USA 111, 2722–2727. (10.1073/pnas.1317454111) 24550301 PMC3932915

[13] Halstead SB, Mahalingam S, Marovich MA, Ubol S, Mosser DM 2010 Intrinsic antibody-dependent enhancement of microbial infection in macrophages: disease regulation by immune complexes. Lancet Infect. Dis. 10, 712–722. (10.1016/S1473-3099(10)70166-3) 20883967 PMC3057165

[14] Dejnirattisai W et al. 2016 Dengue virus sero-cross-reactivity drives antibody-dependent enhancement of infection with zika virus. Nat. Immunol. 17, 1102–1108. (10.1038/ni.3515) 27339099 PMC4994874

[15] Katzelnick LC, Gresh L, Halloran ME, Mercado JC, Kuan G, Gordon A, Balmaseda A, Harris E 2017 Antibody-dependent enhancement of severe dengue disease in humans. Science 358, 929–932. (10.1126/science.aan6836) 29097492 PMC5858873

[16] Nisalak A et al. 2003 Serotype-specific dengue virus circulation and dengue disease in Bangkok, Thailand from 1973 to 1999. Am. J. Trop. Med. Hyg. 68, 191–202. (10.4269/ajtmh.2003.68.191) 12641411

[17] Gibbons RV, Kalanarooj S, Jarman RG, Nisalak A, Vaughn DW, Endy TP, Mammen MP, Srikiatkhachorn A 2007 Analysis of repeat hospital admissions for dengue to estimate the frequency of third or fourth dengue infections resulting in admissions and dengue hemorrhagic fever, and serotype sequences. Am. J. Trop. Med. Hyg. 77, 910–913. (10.4269/ajtmh.2007.77.910) 17984352

[18] Burke DS, Nisalak A, Johnson DE, Scott RM 1988 A prospective study of dengue infections in Bangkok. Am. J. Trop. Med. Hyg. 38, 172–180. (10.4269/ajtmh.1988.38.172) 3341519

[19] Vannice KS, Durbin A, Hombach J 2016 Status of vaccine research and development of vaccines for dengue. Vaccine 34, 2934–2938. (10.1016/j.vaccine.2015.12.073) 26973072

[20] Shrivastava A, Tripathi NK, Dash PK, Parida M 2017 Working towards dengue as a vaccine-preventable disease: challenges and opportunities. Expert Opin. Biol. Ther. 17, 1193–1199. (10.1080/14712598.2017.1356284) 28707486

[21] Hadinegoro SR et al. 2015 Efficacy and long-term safety of a dengue vaccine in regions of endemic disease. N. Engl. J. Med. 373, 1195–1206. (10.1056/NEJMoa1506223) 26214039

[22] Sridhar S et al. 2018 Effect of dengue serostatus on dengue vaccine safety and efficacy. N. Engl. J. Med. 379, 327–340. (10.1056/NEJMoa1800820) 29897841

[23] Wilder-Smith A et al. 2019 Deliberations of the strategic advisory group of experts on immunization on the use of CYD-TDV dengue vaccine. Lancet Infect. Dis. 19, e31–e38. (10.1016/S1473-3099(18)30494-8) 30195995

[24] World Health Organization. 2018 Dengue vaccine: WHO position paper, September 2018—recommendations. Vaccine 87, 317–328. (10.1016/j.vaccine.2018.09.063)

[25] Clapham HE, Wills BA 2018 Implementing a dengue vaccination programme—who, where and how? Trans. R. Soc. Trop. Med. Hyg. 112, 367–368. (10.1093/trstmh/try070) 30016491 PMC6092610

[26] Flasche S et al. 2016 The long-term safety, public health impact, and cost-effectiveness of routine vaccination with a recombinant, live-attenuated dengue vaccine (Dengvaxia): a model comparison study. PLoS Med. 13, e1002181 (10.1371/journal.pmed.1002181) 27898668 PMC5127514

[27] Ferguson NM, Rodriguez-Barraquer I, Dorigatti I, Mier-y-Teran-Romero L, Laydon DJ, Cummings DAT 2016 Benefits and risks of the Sanofi-Pasteur dengue vaccine: modeling optimal deployment. Science 353, 1033–1036. (10.1126/science.aaf9590) 27701113 PMC5268127

[28] Katzelnick LC et al. 2018 Dynamics and determinants of the force of infection of dengue virus from 1994 to 2015 in Managua, Nicaragua. Proc. Natl Acad. Sci. USA 115, 10 762–10 767. (10.1073/pnas.1809253115) PMC6196493 30266790

[29] Rodríguez-Barraquer I et al. 2015 The hidden burden of dengue and chikungunya in Chennai, India. PLoS Negl. Trop. Dis. 9, e0003906 (10.1371/journal.pntd.0003906) 26181441 PMC4504702

[30] Imai N, Dorigatti I, Cauchemez S, Ferguson NM 2015 Estimating dengue transmission intensity from sero-prevalence surveys in multiple countries. PLoS Negl. Trop. Dis. 9, e0003719 (10.1371/journal.pntd.0003719) 25881272 PMC4400108

[31] World Health Organization. 2017 *Informing vaccination programs: a guide to the design and conduct of dengue serosurveys*. http://www.who.int/about/licensing.

[32] Marrero-Santos KM, Beltrán M, Carrión-Lebrón J, Sanchez-Vegas C, Hamer DH, Barnett ED, Santiago LM, Hunsperger EA 2013 Optimization of the cut-off value for a commercial anti-dengue virus IgG immunoassay. Clin. Vaccine Immunol. 20, 358–362. (10.1128/CVI.00429-12) 23302742 PMC3592354

[33] Capeding MR et al. 2014 Clinical efficacy and safety of a novel tetravalent dengue vaccine in healthy children in Asia: a phase 3, randomised, observer-masked, placebo-controlled trial. Lancet 384, 1358–1365. (10.1016/S0140-6736(14)61060-6) 25018116

[34] Cuong HQ, Hien NT, Duong TN, Phong TV, Cam NN, Farrar J, Nam VS, Thai KTD, Horby P 2011 Quantifying the emergence of dengue in Hanoi, Vietnam: 1998–2009. PLoS Negl. Trop. Dis. 5, e1322 (10.1371/journal.pntd.0001322) 21980544 PMC3181236

[35] Cuong HQ et al. 2013 Spatiotemporal dynamics of dengue epidemics, southern Vietnam. Emerg. Infect. Dis. 19, 945–953. (10.3201/eid1906.121323) 23735713 PMC3713821

[36] Quyen DL et al. 2018 Epidemiological, serological, and virological features of dengue in Nha Trang City, Vietnam. Am. J. Trop. Med. Hyg. 98, 402–409. (10.4269/ajtmh.17-0630) 29313471 PMC5929208

[37] Halstead SB et al. 2002 Dengue hemorrhagic fever in infants: research opportunities ignored. Emerg. Infect. Dis. 8, 1474–1479. (10.3201/eid0812.020170) 12498666 PMC2738509

[38] Cuong HQ et al. 2016 Synchrony of dengue incidence in Ho Chi Minh City and Bangkok. PLoS Negl. Trop. Dis. 10, e0005188 (10.1371/journal.pntd.0005188) 28033384 PMC5199033

[39] van Panhuis WG et al. 2015 Region-wide synchrony and traveling waves of dengue across eight countries in Southeast Asia. Proc. Natl Acad. Sci. USA 112, 13 069–13 074. (10.1073/pnas.1501375112) PMC4620875 26438851

[40] Turner HC, Wills BA, Rahman M, Quoc Cuong H, Thwaites GE, Boni MF, Clapham HE 2018 Projected costs associated with school-based screening to inform deployment of Dengvaxia: Vietnam as a case study. Trans. R. Soc. Trop. Med. Hyg. 112, 369–377. (10.1093/trstmh/try057) 29982700 PMC6092611

[41] World Health Organization. 1997 Kinetics of dengue virus replication and host response. In Dengue haemorrhagic fever: diagnosis, treatment, prevention and control, pp. 34–36. Geneva, Switzerland: WHO.

[42] Boni MF et al. 2013 Population-level antibody estimates to novel influenza A/H7N9. J. Infect. Dis. 208, 554–558. (10.1093/infdis/jit224) 23687225 PMC3719906

[43] Nhat NTD et al. 2017 Structure of general-population antibody titer distributions to influenza A virus. Sci. Rep. 7, 6060 (10.1038/s41598-017-06177-0) 28729702 PMC5519701

[44] Berto A et al. 2018 Hepatitis E in southern Vietnam: seroepidemiology in humans and molecular epidemiology in pigs. Zoonoses Public Health 65, 43–50. (10.1111/zph.12364) 28598034 PMC6645987

[45] Quan TM et al. 2018 Evidence of previous but not current transmission of chikungunya virus in southern and central Vietnam: results from a systematic review and a seroprevalence study in four locations. PLoS Negl. Trop. Dis. 12, e0006246 (10.1371/journal.pntd.0006246) 29425199 PMC5823466

[46] Alere Inc. 2016 Panbio® Dengue IgG indirect ELISA. http://www.alere.com/en/home/product-details/panbio-dengue-igg-indirect-elisa.html (accessed 23 November 2018).

[47] Ferguson NM, Donnelly CA, Anderson RM 1999 Transmission dynamics and epidemiology of dengue: insights from age-stratified sero-prevalence surveys. Phil. Trans. R. Soc. Lond. B 354, 757–768. (10.1098/rstb.1999.0428) 10365401 PMC1692557

[48] R Development Core Team. 2008 R: a language and environment for statistical computing. Vienna, Austria: R Foundation for Statistical Computing http://www.r-project.org.

[49] Cummings DAT, Iamsirithaworn S, Lessler JT, McDermott A, Prasanthong R, Nisalak A, Jarman RG, Burke DS, Gibbons RV 2009 The impact of the demographic transition on dengue in Thailand: insights from a statistical analysis and mathematical modeling. PLoS Med. 6, e1000139 (10.1371/journal.pmed.1000139) 19721696 PMC2726436

[50] University of Pittsburgh. 2019 Project Tycho. https://www.tycho.pitt.edu/data.

[51] Tien NTK, Luxemburger C, Toan NT, Pollissard-Gadroy L, Huong VTQ, Van Be P, Rang NN, Wartel T-A, Lang J 2010 A prospective cohort study of dengue infection in schoolchildren in Long Xuyen, Vietnam. Trans. R. Soc. Trop. Med. Hyg. 104, 592–600. (10.1016/j.trstmh.2010.06.003) 20630553

[52] Imai N, Dorigatti I, Cauchemez S, Ferguson NM 2016 Estimating dengue transmission intensity from case-notification data from multiple countries. PLoS Negl. Trop. Dis. 10, e0004833 (10.1371/journal.pntd.0004833) 27399793 PMC4939939

[53] Kucharski AJ et al. 2018 Using paired serology and surveillance data to quantify dengue transmission and control during a large outbreak in Fiji. Elife 7, 1–26. (10.7554/eLife.34848) PMC6092126 30103854

[54] Rodríguez-Barraquer I, Salje H, Cummings DA 2019 Dengue pre-vaccination screening and positive predictive values. Lancet Infect. Dis. 19, 132–134. (10.1016/s1473-3099(18)30799-0) 30712834

