# Supplementary material for: Serological inference of past primary and secondary dengue infection: implications for vaccination
Source: J R Soc Interface. 2019 Jul 31;16(156):20190207. doi: 10.1098/rsif.2019.0207 (PMC6685028; doi:10.1098/rsif.2019.0207)
Supplement: Supplementary Information for Lam et al. (2019) [file rsif20190207supp1.pdf]

SUPPLEMENTARY METHODS

BUILDING THE CONTINUOUS MODELS

Let E_i denote the number of dengue serotypes by which individual i has been challenged. According to the catalytic model (Ferguson, Donnelly and Anderson 1999), the probability that the individual is still naïve to dengue – given his age, a – is $P(E_i = 0|a_i) = \exp(-\bar{E}_i)$, in which $\bar{E}_i$ is calculated as follows:

$$\bar{E}_i = \sum_{u=1}^{p} \mathbf{1}[\kappa_u < a_i] \times (\kappa_u - \kappa_{u-1}) \times \lambda_{\kappa_{u-1},\kappa_u} + \left[a_i - \max_{\kappa_v}(\kappa_v < a_i)\right] \times \lambda_{\kappa_v,\kappa_{v+1}}$$

where:

a_i is the age (in years) of individual i;

$\lambda_{\kappa_{u-1},\kappa_u}$ is the total annual FOI of all the four serotypes of dengue that the age group between κ_{u-1} and κ_u years are exposed to;

κ_u, κ_{u-1} are age cut-offs ($\kappa_0 = 0$ and $\kappa_p = \infty$);

$p \in \{1, 2, 3\}$ is the number of age-specific FOIs in the considered model;

$\mathbf{1}[\kappa_u < a_i]$ is an indicator function, i.e. $\mathbf{1}[\kappa_u < a_i] = \begin{cases} 1 & \text{if } \kappa_u < a_i \\ 0 & \text{otherwise} \end{cases}$.

The probability that individual i has experienced at least one dengue infection is:

$$P(E_i \geq 1|a_i) = 1 - P(E_i = 0|a_i) = 1 - \exp(-\bar{E}_i)$$

If we assume that all four dengue serotypes have equal FOIs (i.e. $\frac{\lambda}{4}$), the probability that an individual has been challenged by one of the four serotypes but is still naïve to the other serotypes can be calculated as follows:

$$P(E_i = 1|a_i) = 4 \times \exp\left(-\frac{3}{4}\bar{E}_i\right) \times \left(1 - \exp\left(-\frac{1}{4}\bar{E}_i\right)\right)$$

where $\left(1-\exp\left(-\frac{1}{4}\bar{E}_i\right)\right)$ is the probability that the individual has experienced one dengue serotype, and $\exp\left(-\frac{3}{4}\bar{E}_i\right)$ corresponds to the chance that the individual has escaped the other three serotypes.

The probability that an individual has been exposed to more than one dengue serotype is:

$$P(E_i \geq 2|a_i) = 1 - P(E_i = 0|a_i) - P(E_i = 1|a_i) = 1 - e^{-\bar{E}_i} - 4e^{-\frac{3}{4}\bar{E}_i}\left(1 - e^{-\frac{1}{4}\bar{E}_i}\right)$$

Assume that the IgG antibody level of each individual, Y_i, follows a gamma distribution that is conditional on the number of dengue exposures (E_i). In models with two distributions of IgG levels (i.e. $Y_i|E_i = 0$ and $Y_i|E_i \geq 1$), the binomial likelihoods of the models were calculated as follows:

$$\begin{aligned}\mathcal{L}(\boldsymbol{\theta}|\boldsymbol{Y}) &\propto \prod_{i=1}^{n}[f(Y_i|E_i = 0) \times P(E_i = 0|a_i) + f(Y_i|E_i \geq 1) \times P(E_i \geq 1|a_i)] \\ &= \prod_{i=1}^{n}\left[f(Y_i|E_i = 0) \times e^{-\bar{E}_i} + f(Y_i|E_i \geq 1) \times \left(1 - e^{-\bar{E}_i}\right)\right]\end{aligned}$$

where $f(Y_i|E_i = 0)$ and $f(Y_i|E_i \geq 1)$ are the probability density functions of IgG levels of seronegative and seropositive individuals, respectively.

Similarly, in models with three IgG-level distributions (i.e. $Y_i|E_i = 0$, $Y_i|E_i = 1$, and $Y_i|E_i \geq 2$), the multinomial likelihoods were calculated as follows:

$$\begin{aligned}\mathcal{L}(\boldsymbol{\theta}|\boldsymbol{Y}) \propto \prod_{i=1}^{n}&[f(Y_i|E_i = 0) \times P(E_i = 0|a_i) + f(Y_i|E_i = 1) \times P(E_i = 1|a_i) \\ &+ f(Y_i|E_i \geq 2) \times P(E_i \geq 2|a_i)] \\ &= \prod_{i=1}^{n}\left[f(Y_i|E_i = 0) \times e^{-\bar{E}_i} + f(Y_i|E_i = 1) \times 4e^{-\frac{3}{4}\bar{E}_i}\left(1 - e^{-\frac{1}{4}\bar{E}_i}\right)\right. \\ &\left.+ f(Y_i|E_i \geq 2) \times \left(1 - e^{-\bar{E}_i} - 4e^{-\frac{3}{4}\bar{E}_i}\left(1 - e^{-\frac{1}{4}\bar{E}_i}\right)\right)\right]\end{aligned}$$

where $f(Y_i|E_i = 0)$, $f(Y_i|E_i = 1)$ and $f(Y_i|E_i \geq 2)$ are the probability density functions of IgG levels of individuals given that they were estimated to be seronegative (naïve to dengue), primary-seropositive (having experienced only primary infections), or secondary-seropositive (having experienced secondary infections), respectively.

In these models, monotypic re-exposure was assumed not to alter IgG antibody levels.

SUPPLEMENTARY RESULTS

Table S1: *Fifteen binary models that were fitted to the data of An Giang, Ho Chi Minh City, and Quang Ngai separately. Models are arranged by their deviance information criterion (DIC) in ascending order. DIC scores and median estimates of the force of infection (with 95% credible intervals) are only shown for converged models.*

	Model	No. Free Param.	Age Groups Count	Age Groups Cut-offs[1]	Force of Infection[2]			DIC
An Giang	AgiBin02	2	2	6	0.06 (0.04; 0.09)		0.17 (0.13; 0.21)	206
An Giang	AgiBin01	1	1	-		0.11 (0.09; 0.13)		218
An Giang	AgiBin03	3	2	est.		-		-
An Giang	AgiBin04	3	3	6 and 18		-		-
An Giang	AgiBin05	5	3	est.		-		-
Ho Chi Minh City	HcmBin01	1	1	-		0.07 (0.06; 0.09)		387
Ho Chi Minh City	HcmBin02	2	2	6	0.08 (0.05; 0.12)		0.07 (0.05; 0.10)	389
Ho Chi Minh City	HcmBin04	3	3	6 & 18	0.07 (0.04; 0.12)	0.08 (0.05; 0.12)	0.05 (0.00; 0.12)	390
Ho Chi Minh City	HcmBin03	3	2	est.		-		-
Ho Chi Minh City	HcmBin05	5	3	est.		-		-
Quang Ngai	QngBin01	1	1	-		0.03 (0.02; 0.04)		331
Quang Ngai	QngBin02	2	2	6	0.04 (0.02; 0.07)		0.02 (0.01; 0.03)	331
Quang Ngai	QngBin04	3	3	6 & 18	0.05 (0.03; 0.07)	0.02 (0.00; 0.04)	0.03 (0.01; 0.05)	332
Quang Ngai	QngBin03	3	2	est.		-		-
Quang Ngai	QngBin05	5	3	est.		-		-

1 *"est." indicates that the parameters were left to be estimated by the MCMC runs, but the chains did not converge.*

2 *The number of FOI estimates in each model corresponds to the number of age groups in the model, with the first estimate corresponding to the youngest age group.*

Table S2: *Configuration of the IgG-level distributions in the four continuous models that were fitted to the whole Vietnamese data set. In these models, we assumed constant FOIs for Ho Chi Minh City and Quang Ngai, and an age-varying FOI for An Giang (with two age groups). Models are arranged by their deviance information criterion (DIC) in ascending order.*

Model	Configuration of the IgG-level Distributions		No. Free Param.	DIC
	Seronegative	Primary-seropositive & Secondary-seropositive		
VnmCon01	population-specific	population-specific	20	6095
VnmCon02	shared across populations	population-specific	18	6140
VnmCon03	population-specific	shared across populations	12	6175
VnmCon04	shared across populations	shared across populations	10	6196

Table S3: *Six continuous-titre models that were fitted to the Chennai dataset. The posteriors of the IgG-level distributions of seronegative and primary-seropositive cases of An Giang (inferred from the AgiCon33 model) were used as strong priors for the corresponding IgG-level distributions in these models of Chennai. The models are arranged by their deviance information criterion (DIC) in ascending order; the last models in the list did not converge. Estimated parameters are shown by the median values and their 95% credible intervals (in parentheses). Fixed parameters are presented as a single number. The heterogeneity of the FOI (if it exists) is interpreted as being time-dependent as suggested by the original paper.*

Model (DIC)	No. Free Param.	Time Periods		Force of Infection[2]			Mean & Std. Dev. of Sero-negative	Primary-seropositive		Secondary-seropositive	
		Count	Cut-offs[1]	1st Time Period	2nd Time Period	3rd Time Period		Mean	Std. Dev.	Mean	Std. Dev.
CheCon32e (5,565)	7	2	7	0.28 (0.23; 0.33)	0.06 (0.04; 0.08)	-	4.8 (3.7; 6.1)	32.3 (30.0; 34.4)	9.2 (7.9; 10.8)	41.4 (40.9; 41.9)	4.6 (4.3; 5.0)
CheCon33 (5,565)	8	2	9.4 (6.4; 13.3)	0.24 (0.19; 0.31)	0.05 (0.02; 0.07)	-	4.7 (3.7; 6.1)	32.3 (30.0; 34.3)	9.2 (7.9; 10.8)	41.4 (40.9; 41.9)	4.6 (4.3; 5.0)
CheCon32 (5,568)	7	2	6	0.30 (0.25; 0.36)	0.06 (0.04; 0.08)	-	4.8 (3.7; 6.1)	32.4 (30.0; 34.4)	9.3 (8.0; 10.8)	41.4 (40.9; 41.9)	4.6 (4.3; 5.0)
CheCon34 (5,568)	8	3	6 & 18	0.28 (0.22; 0.35)	0.09 (0.04; 0.14)	0.05 (0.01; 0.08)	4.7 (3.7; 6.1)	32.3 (29.9; 34.3)	9.3 (7.9; 10.8)	41.4 (40.9; 41.9)	4.7 (4.3; 5.0)
CheCon31 (5,621)	6	1	-	0.14 (0.12; 0.15)	-	-	5.0 (3.9; 6.5)	33.5 (31.2; 35.3)	9.5 (8.2; 11.0)	41.2 (40.7; 41.7)	4.8 (4.4; 5.3)
CheCon35 (N/A)	10	3	est.	-	-	-	-	-	-	-	-

1 *"est." indicates that the time cut-offs were left to be estimated by the MCMC runs, but the chains did not converge.*

2 *The number of FOI estimates in each model corresponds to the number of time periods in the model, with the first estimate corresponding to the most recent time period before the year of sample collection (2011).*

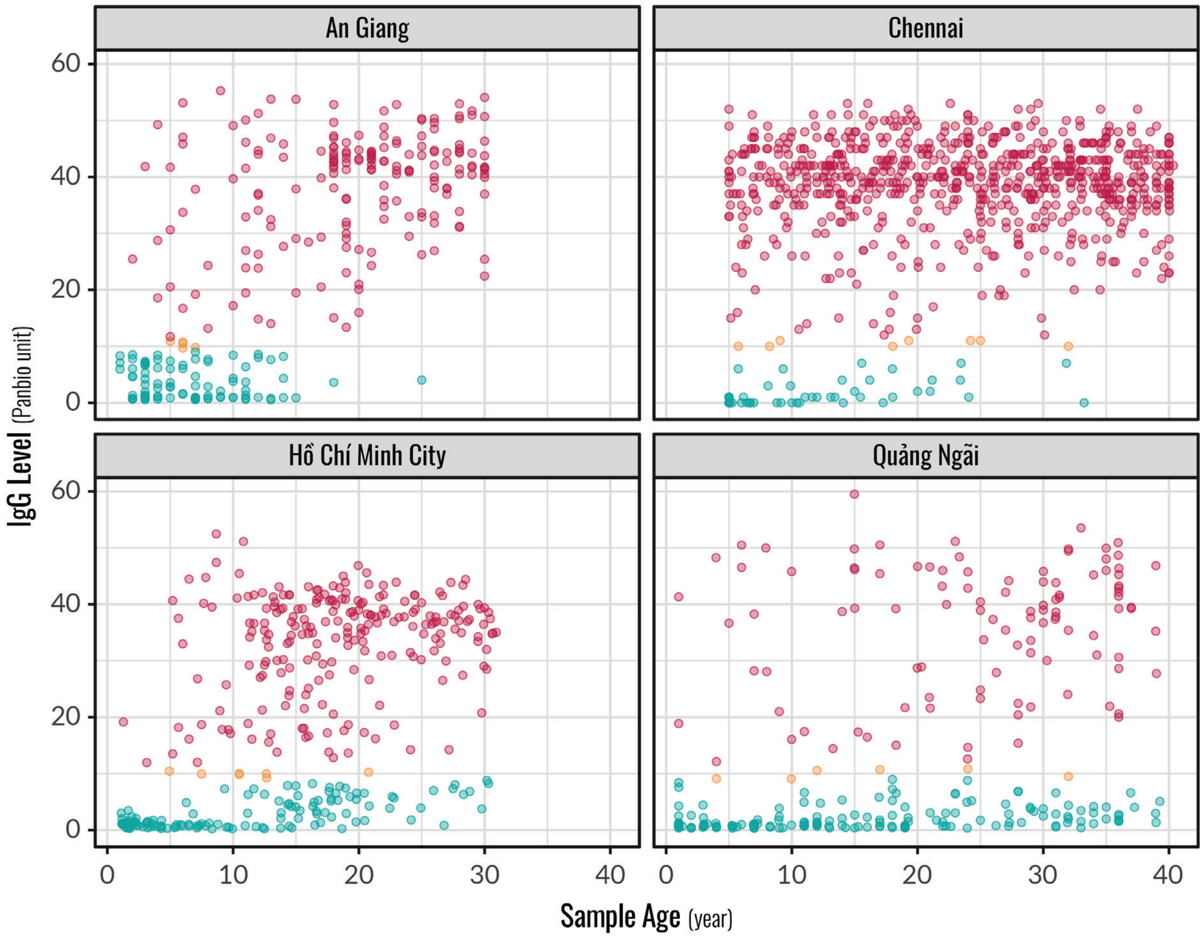

Figure S1: *The ages and the IgG titres of the samples used in this analysis. Teal, orange, and red points represent seronegative (IgG titre < 9), equivocal (9 ≤ IgG titre ≤ 11), and seropositive (IgG titre > 11) samples, respectively.*

IgG Level (Panbio unit)

Age (year)	1	3	5	7	9	11	13	15	17	19	21	23	25	27	29	31	33	35	37	39	41	43	45
1	0	1	8	26	55	80	92	97	99	100	100	100	100	99	96	90	80	70	64	61	62	67	74
4	0	6	26	60	84	94	98	99	100	100	100	100	99	95	84	66	47	35	28	26	27	31	39
7	1	10	39	73	91	97	99	100	100	100	100	100	98	91	73	49	31	21	17	15	16	19	25
10	1	14	49	80	93	98	99	100	100	100	100	100	97	87	63	38	22	14	11	10	10	12	17
13	1	18	57	85	95	98	99	100	100	100	100	100	96	81	53	29	16	10	8	7	7	9	12
16	2	22	63	88	96	99	100	100	100	100	100	99	94	76	45	22	12	7	6	5	5	6	9
19	2	27	68	90	97	99	100	100	100	100	100	99	93	70	37	17	9	6	4	4	4	5	7
22	2	31	72	92	97	99	100	100	100	100	100	99	90	63	31	14	7	4	3	3	3	4	5
25	3	35	76	93	98	99	100	100	100	100	100	98	88	56	25	11	5	3	2	2	2	3	4
28	3	38	79	94	98	99	100	100	100	100	100	98	84	49	20	8	4	2	2	2	2	2	3
31	4	42	81	95	98	100	100	100	100	100	100	97	80	43	16	7	3	2	1	1	1	2	2
34	4	46	83	95	99	100	100	100	100	100	100	96	76	37	13	5	2	1	1	1	1	1	2
37	5	49	85	96	99	100	100	100	100	100	100	95	71	31	10	4	2	1	1	1	1	1	1
40	6	52	87	96	99	100	100	100	100	100	100	94	65	26	8	3	1	1	1	1	1	1	1
43	6	56	88	97	99	100	100	100	100	100	99	92	59	21	6	2	1	1	1	0	0	1	1
46	7	59	89	97	99	100	100	100	100	100	99	90	53	17	5	2	1	1	0	0	0	0	1
49	8	61	90	98	99	100	100	100	100	100	99	88	47	14	4	1	1	0	0	0	0	0	0

Hồ Chí Minh

Probability of being **primary-seropositive** (%): 0 – 25 – 50 – 75 – 100

Figure S2: *The probability of a given sample in Ho Chi Minh City being primary-seropositive. This probability table was built based on the IgG-level distributions and the median FOI estimate of Ho Chi Minh City, inferred from the HcmCon31 model. The blue area on the left side of the table represents individuals who are likely to be seronegative. Meanwhile, individuals falling into the blue area on the right side are likely to be secondary-seropositive.*

IgG Level (Panbio unit)

Age (year)	1	3	5	7	9	11	13	15	17	19	21	23	25	27	29	31	33	35	37	39	41	43	45
1	0	0	2	7	18	38	62	81	91	96	98	99	100	100	100	99	98	96	93	90	88	86	86
4	0	2	8	22	47	71	87	94	98	99	100	100	100	100	98	96	91	84	76	68	63	60	60
7	0	3	13	34	61	81	92	97	99	99	100	100	100	99	97	93	84	74	63	54	48	45	45
10	0	5	18	43	70	86	94	98	99	100	100	100	100	99	96	89	78	65	53	44	38	36	35
13	1	6	23	50	75	89	96	98	99	100	100	100	100	98	95	86	73	58	45	36	31	29	29
16	1	8	27	56	79	91	97	99	99	100	100	100	100	98	93	83	67	51	39	31	26	24	24
19	1	9	31	60	82	93	97	99	100	100	100	100	99	98	92	79	62	46	34	26	22	20	20
22	1	10	34	64	84	94	98	99	100	100	100	100	99	97	90	76	57	41	29	23	19	17	17
25	1	12	38	68	86	95	98	99	100	100	100	100	99	96	88	72	53	37	26	20	16	15	15
28	1	13	41	70	88	95	98	99	100	100	100	100	99	96	86	69	49	33	23	17	14	13	13
31	2	15	44	73	89	96	98	99	100	100	100	100	99	95	84	65	45	29	20	15	12	11	11
34	2	16	47	75	90	96	99	99	100	100	100	100	99	94	82	62	41	26	18	13	11	10	10
37	2	18	49	77	91	97	99	99	100	100	100	100	99	94	80	58	38	24	16	12	9	8	8
40	2	19	52	79	92	97	99	100	100	100	100	100	98	93	78	55	34	21	14	10	8	7	7
43	2	21	54	80	92	97	99	100	100	100	100	100	98	92	75	52	32	19	13	9	7	7	7
46	3	22	56	82	93	97	99	100	100	100	100	100	98	91	73	49	29	17	11	8	7	6	6
49	3	24	58	83	94	98	99	100	100	100	100	100	98	90	70	45	26	16	10	7	6	5	5

Quảng Ngãi

Probability of being **primary-seropositive** (%): 0 – 25 – 50 – 75 – 100

Figure S3: *The probability of a given sample in Quang Ngai being primary-seropositive. This probability table was built based on the IgG-level distributions and the median FOI estimate of Quang Ngai, inferred from the HcmCon31 model. The blue area on the left side of the table represents individuals who are likely to be seronegative. Meanwhile, individuals falling into the blue area on the right side are likely to be secondary-seropositive.*

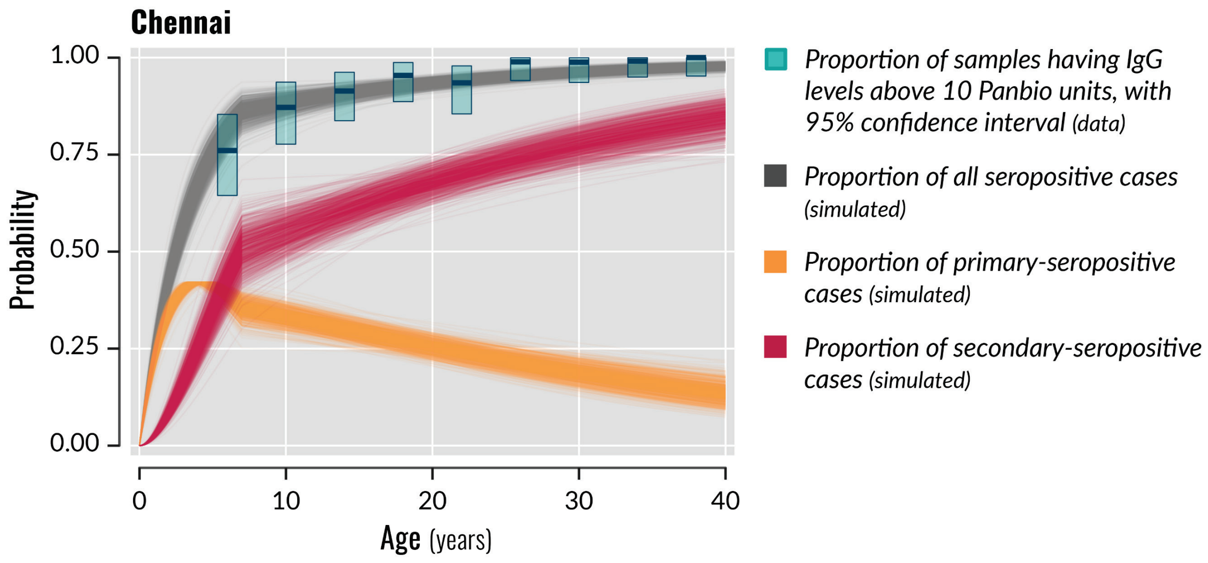

Figure S4: The seroprevalence of dengue in Chennai, India. The bars represent the proportion (with 95% confidence intervals) of samples for each 4-year age group with IgG levels above 10 Panbio units. The lines show the estimates of seroprevalence from 1,000 simulations, of which the parameters were drawn from the posteriors of the CheCon32e model.

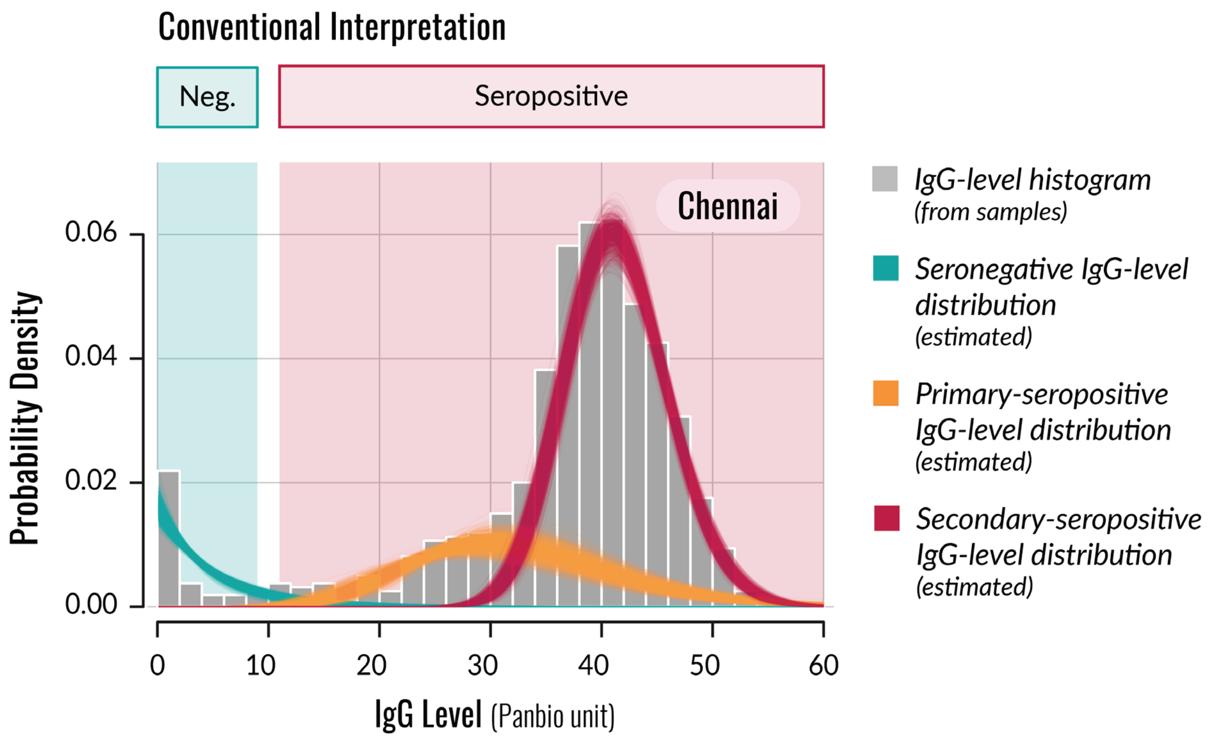

Figure S5: The histogram of the measured IgG levels (bars), and the inferred IgG-level distributions (lines) of the three exposure classes (seronegative, primary-seropositive, secondary-seropositive) in Chennai. The IgG-level distributions were simulated 1,000 times based on the posteriors of the CheCon32e model.
